# Rush Medical College

**Published:** 1867-03

**Authors:** 


*Rush Medical College—A Voice from Cincinnati.*—The little city opposite Covington, Kentucky, or rather that portion of it which, at the present writing, is above the muddy waters of the Ohio, has an always present, abiding anxiety about the welfare of Chicago—the metropolis of the North-west. Its secular press eagerly collects the garbage of the sensational reporters and items-men of the Chicago press, and retails it with a Pharisaical unction, delightful to contemplate. The untidy hamlet which the Ohio is just now vainly attempting to cleanse, has seen the sceptre of commercial supremacy pass from its trembling digits to the strong hands of this great city, and it mumbles its impotent wrath like a toothless grandam enraged at the loss of her juvenile charms. Among its later efforts in this direction we notice that it has made use of the mercurial intellect of "our Hibernian friend" of that generally dignified and courteous professional magazine, the *Lancet and Observer*. Our Celtic contemporary has had his senses sharpened by some gale from the North-west, and, like his lamented countryman, Sir Boyle Roche, 'he smells a rat, — he sees it floating in the air,' and all about Rush Medical College. He is as melancholy over it as a salivated patient at the sight of a generous dinner—(a simile, by the by, germain to Cincinnati if not Celtic.) Our lachrymose friend writes thus:—

"We regret to see our esteemed neighbors of the Rush College thus making so light a matter of a course of instruction. "The present session could scarcely have afforded three months "of lecture term! Are students and practitioners willing to "encourage such a plan of instruction? The age and influence "of this prominent school of the North-west should warrant it "in taking a very high stand in all that pertains to granting "the doctorate."

This journal benevolently rejoices in the opportunity of dissipating the melancholy which hangs around the lugubrious brow of our esteemed cotemporary.

The last session of Rush Medical College commenced Wednesday, the third day of October, 1866, and concluded Wednesday, the thirtieth day of January, 1867. An arithmetician of moderate ability can estimate the interval between these dates. If there are none in Cincinnati who can make this more than "scarcely three months of lecture term," it is advised that they borrow elasticity from their consciences and add to their intellects. As a matter of fact, the term extended to seventeen weeks, the extra week being occupied in consequence of the appropriate recess, taken on account of the sudden demise of Prof. Brainard. The usual number of lectures was given during the last session—the only loss in time to the students being two working days, which was more than made up by extra lectures during the balance of the session. As a matter of fact, no medical college in this country gave, or has at any time given, more lectures in the session of sixteen weeks than were given by the Faculty of Rush Medical College during the session of 1866 and 1867.

Are there two Dromios in this Cincinnati Comedy of Errors, or does "Our Hibernian Friend" also play the part of correspondent (melancholy again) to Prof. Gross? *Item:* "The classes are *all* small at St. Louis and Chicago."—(*Lancet and Observer*, Feb., 1867, p. 109.) Like information gained during the war from the "intelligent contraband" and "reliable gentleman," this is news, "important if true." So far as Rush Medical College is concerned, this journal begs leave to inform both Prof. Gross and his correspondent, that there was no falling off in attendance at this College, notwithstanding the derangement produced temporarily by the death of Prof. Brainard. The class, the last session, as during several previous sessions, has only been limited in numbers by the capacity of the College lecture rooms to furnish, not merely seats, but standing room. On the opening of the new college edifice, with its magnificent lecture rooms, and well-arranged and commodious apartments, next October, another onslaught upon the institution may be predicted, from a point upon the Ohio bottoms, which need not be more particularly specified. Until then, Cincinnati, *vale, et cura cuticulam tuam.*

